# A Deep Convolutional Neural Network Method to Detect Seizures and Characteristic Frequencies Using Epileptic Electroencephalogram (EEG) Data

**DOI:** 10.1109/JTEHM.2021.3050925

**Published:** 2021-01-11

**Authors:** Md. Rashed-Al-Mahfuz, Mohammad Ali Moni, Shahadat Uddin, Salem A. Alyami, Matthew A. Summers, Valsamma Eapen

**Affiliations:** 1 Department of Computer Science and EngineeringUniversity of Rajshahi 118869 Rajshahi6205Bangladesh; 2 Faculty of Medicine, School of PsychiatryUniversity of New South Wales 7800 SydneyNSW2052Australia; 3 Complex Systems Research Group, Faculty of EngineeringThe University of Sydney 4334 DarlingtonNSW2008Australia; 4 Department of Mathematics and StatisticsImam Mohammad Ibn Saud Islamic UniversityRiyadh11432Saudi Arabia; 5 Garvan Institute of Medical Research 2785 DarlinghurstNSW2010Australia

**Keywords:** Epilepsy, seizure, EEG, deep learning, CWT, STFT

## Abstract

Background: Diagnosing epileptic seizures using electroencephalogram (EEG) in combination with deep learning computational methods has received much attention in recent years. However, to date, deep learning techniques in seizure detection have not been effectively harnessed due to sub-optimal classifier design and improper representation of the time-domain signal. Methods: In this study, we focused on designing and evaluating deep convolutional neural network-based classifiers for seizure detection. Signal-to-image conversion methods are proposed to convert time-domain EEG signal to a time-frequency represented image to prepare the input data for classification. We proposed and evaluated three classification methods comprising of five classifiers to determine which is more accurate for seizure detection. Accuracy data were then compared to previous studies of the same dataset. Results: We found our proposed model and signal-to-image conversion method outperformed all previous studies in the most cases. The proposed FT-VGG16 classifier achieved the highest average classification accuracy of 99.21%. In addition, the Shapley Additive exPlanations (SHAP) analysis approach was employed to uncover the feature frequencies in the EEG that contribute most to improved classification accuracy. To the best of our knowledge, this is the first study to compute the contribution of frequency components to target seizure classification; thus allowing the identification of distinct seizure-related EEG frequency components compared to normal EEG measures. Conclusion: Thus our developed deep convolutional neural network models are useful to detect seizures and characteristic frequencies using EEG data collected from the patients and this model could be clinically applicable for the automated seizures detection.

## I. Introduction

Epilepsy is a chronic neurological disorder that affects approximately 50 million people worldwide, with around 2.4 million people newly diagnosed annually [1]. Electroencephalogram (EEG) is a widely-used non-invasive technique for the measurement of brain electrical activity and the diagnosis of epilepsy. The analysis and interpretation of EEG data are usually made by manual visual inspection by neurologists. However, visual examination of EEG traces is time-consuming and puts a heavy burden on the treating physician. These issues have inspired significant efforts in the application of automated seizure detection techniques to assist neurologists, speed up the diagnosis process and, thereby, improve the accuracy. Additionally, studying the frequency features in EEG seizure data is important to progress our fundamental understanding of seizure EEG traces.

The application of machine learning towards automatic seizure detection is reported in many studies. For example, several shallow machine learning techniques, including neural systems and Support Vector Machine (SVM) methods have been used for epilepsy classification [2]; however, there remains a need to develop improved algorithms achieving higher classification accuracy, so that automated artificial intelligent systems and tools can be developed for clinical applications.

There is a considerable demand to extend the applications of machine learning, especially the emerging domain of deep learning, to the classification of EEG signals in automated seizure detection. The application of deep learning for disease diagnosis are growing in general, and several studies have been published, but with limited performance [3]–[5]. Recently, studies have been conducted on the specific classification problem using machine and deep learning techniques to identify epileptic and non-epileptic EEG signals [6]–[14]. However, there remains significant room for improvement of deep learning based methods for automated disease classification.

A recent study on seizure detection used a 13-layer deep 1D CNN and the University of Bonn database, achieving 88.67% classification accuracy [9]. The CNN based model has also been used in epileptic seizure detection using the Freiburg and CHB-MIT databases, achieving high precision outcomes of 96.7 percent and 97.5 percent, respectively [15]. In another study, a pyramidal 1-dimensional CNN model for binary (seizure vs. non-seizure) classification using the University of Bonn database has been proposed [7]. Using a deep CNN model on the TUH database, a sensitivity of 30.83% and specificity of 96.86% was achieved [16]. Besides CNN based models, many studies used entropy-based features of EEG in seizure detection [17]–[19]. SVM and multilayer perceptron were also used in epileptic seizure detection [17], [20].

With the goal of improved results, some studies used spectral images of the EEG signal with a CNN-based classifier. Bi and Wang [21] used spectral images of time domain signal as input to a CNN model for disease diagnosis. One study [22] used Fourier based time-frequency representation using STFT for deep learning based seizure classification, and with VGG16 achieved 79.71% accuracy. Raghu *et al.* [14] proposed deep CNNs and spectrograms of EEG, with the highest accuracy of 84.06% using Temple University Hospital database EEG recordings. Seizure and non-seizure EEG activities have been classified using CNN and plot-EEG-image input and achieved a true positive rate of 74.0% [23]. Image-based representation of EEG spectrograms was used as input to a CNN based classifier for seizure detection [24].

From the studies mentioned above, it is evident that the deep learning models are useful in image-based seizure detection applications. However, to our knowledge, effective deep learning models using time-frequency image data of EEG for seizure detection have not been conducted.

In addition to obtaining high classification accuracy, it is often important to understand the features of the input data that most contributed to the classification. A promising technique called SHAP (SHapley Additive exPlanation) [25] uses shapley values to explain predictions by calculating feature importance. By using SHAP we can understand the significant frequency features of the input time-frequency EEG image to be tested. These features are distinct from the inputs in other classes as each is assigned a value for a particular prediction.

In this study we propose a time-domain-signal to time-frequency-image conversion method using continuous wavelet transform (CWT), to prepare the input data for the deep learning model. We also propose three different classification methods in which a classifier consists of 4 convolutional layers (method-1). Two adopted deep learning models, VGG16 and ResNet50, are used in method-2 and method-3. All the methods are tested with the CWT scalogram and STFT spectrogram. We also used SHAP and a gradient-based model explainer to find characteristic frequencies in EEG seizures that are distinct from normal EEG, and are responsible for achieving the improved classification accuracy.

## II. Materials and Methods

### A. Data

We have used EEG data from the repository of the Bonn University [26]. The full database consists of five sets (A–E) in which each contains 100 single-channel EEG segments with a duration of 23.6 seconds. The muscle activity and eye movement artifacts were already removed from the collected data on the basis of visual inspection. The EEG recording was performed based on standardised electrode placement techniques.

Set A contains surface EEG recordings collected from five healthy subjects in the wakeful state with eyes open. Set B contains EEG acquired during eyes closed from the same subjects. The other datasets (C, D, and E) were collected during the pre-surgical diagnostic work up of five seizure patients. Set C contains EEG recordings that were recorded from the hippocampal formation of opposite hemispheric regions during seizure-free intervals. Set D comprises the EEG signal collected from within the epileptic zone of the brain of patients during seizure-free intervals. The last one (set E) contains EEG recordings of patients during seizure activity. The sampling rate of the EEG signal was 173.61 Hz after 12-bit conversion using a 12-bit A/D converter.

### B. Preprocessing

Although there are many 1D CNN models to classify time-domain signals, 2D CNN models are still important in the classification problems. Since time-domain EEG signals can be transformed into 2D (RGB) images, we choose 2D CNN for the present classification task. We segment the EEG signals into pieces of 1.47 seconds length and then performed signal-to-image conversion on each segment. We also considered the color (RGB) representation of the time-frequency conversion of the EEG signal. The two widely used methods, STFT and CWT were applied in this study.

#### 1) STFT

The short-term Fourier transform (STFT) is one of the widely used methods for the time-frequency analysis [27]. STFT determines the frequency and phase of local sections of a segment of a signal. This segmentation is performed by using a frame. Since the frame shifts over time, STFT functions as a trade-off between a time-based and a frequency-based representation. Thus, STFT is capable of showing frequencies contained in the signal at the corresponding time points. The short segments of signal are taken using a moving window $g\left ({t}\right)$, centered at $u$, and the Fourier transform is performed on those segments. Most commonly, a Hamming window is used for STFT. The STFT is defined for a long time segment $f\left ({t}\right)$ as:

\begin{equation*} Y\left ({{\omega,u}}\right)=STFT\{f{\left ({t}\right)}\}=\int _{R}f\left ({t}\right)g\left ({t-u}\right)e^{-j\omega {t}}dt\tag{1}\end{equation*}

The spectrogram (energy surface distribution of STFT) is computed as:

\begin{equation*} E\left ({{\omega,u}}\right)= {|Y\left ({{\omega,u}}\right)|}^{2}\tag{2}\end{equation*}

We get different spectra at each corresponding time and the totality of these spectra are the spectrogram.

#### 2) CWT

Continuous wavelet transformation (CWT) is considered to be efficient in the time-frequency analysis of the nonstationary signal.

The CWT for a real signal $x\left ({t}\right)\in {L^{2}{\left ({\mathbb {R}}\right)}}$ with translation parameter $\tau \in \mathbb {R}$, scale parameter $s>0$ and wavelet function $\psi \left ({t}\right)$

\begin{equation*} CWT_{x}^{\psi }{\left ({s,\tau }\right)}=\int _{-\infty }^{+\infty }x\left ({t}\right){\frac {1}{\sqrt {s}}}\psi {\left ({{\frac {t-\tau }{s}}}\right)}dt\tag{3}\end{equation*}

Here, $\tau $, the time shift of the translation, can be interpreted as the time instant around which the signal is analyzed.

With small $s$ values, CWT provides detailed information of the signal in the neighbourhood of the instant $\tau $, that is the high frequency content; whereas with large $s$ CWT provides lower frequency content in the neighbourhood of the time instant.

A two-dimensional image, called a scalogram, is used to represent the square of CWT, $|CWT_{x}^{\psi }{\left ({s,\tau }\right)}|^{2}$. Since, the analyzed signal is a digital signal, a discrete approximation of $CWT_{x}^{\psi }{\left ({s,\tau }\right)}$ is computed [28]. A matrix, with rows and columns representing different scales $s$ and translation parameters $\tau $, respectively, is used to visualize the approximated scalogram. However, in time-frequency representation, frequency is more conventional than scale. We converted scale to frequency as $f=\frac {1}{s}$.

### C. Convolutional Neural Networks (CNN)

In general, artificial neural networks (ANN) consist of three layers, namely, input, hidden, and output layers. It is considered as an information processing paradigm that is inspired by the complex network structure of the biological nervous system in the human brain. An ANN is composed of a collection of connected elements called nodes or artificial neurons. Those artificial neurons integrate the input signals coming from other nodes in the preceding layer, and transfer them to neurons in the next layer. The receiving neuron produces its output by summing the weighted signals from all neurons to which it is connected in the preceding layer. In the network, the first is the input layer and the last is considered as the output layer.

This study employs an enhanced and newly developed neural network, known as Convolutional Neural Network (CNN). Basic CNN consists of four types of layers, namely, convolutional, activation, pooling, and fully-connected layers. The convolutional, activation and pooling layer aims to learn feature representations of the inputs, whereas the fourth is a fully connected layer that performs the classification. The non-linear activation layer following the convolutional layers is responsible for capturing more complex properties of the input signal.

Convolution layer consists of several convolution kernels. Each kernel is responsible for computing distinct feature maps. Each neuron of the layer is only connected to a small local area of the preceding layer, which resembles the receptive field in the human visual system. Each layer $l$ has $M$ number of feature maps, each of size $\left ({M_{x}, M_{y}}\right)$. The high-level features are extracted by sliding a kernel of size $\left ({K_{x},K_{y}}\right)$ over the valid region of the input data. The skipping factors $S_{x}$ and $S_{y}$, also called stride size, define how many pixels the filter/kernel skips in x and y direction between subsequent convolutions. The size of the calculated feature map is then defined as:

\begin{align*} M_{x}^{l}=&\frac {{M_{x}^{l-1}-{K_{x}^{l}}}}{S_{x}^{l}+1}+1 \tag{4}\\ M_{y}^{l}=&\frac {{M_{y}^{l-1}-{K_{y}^{l}}}}{S_{y}^{l}+1}+1\tag{5}\end{align*}

where $l$ defines the layer in the network and each feature map in layer $l$ is associated with at most $M^{l-1}$ maps in layer $l-1$. The kernel is shared by all spatial locations of the input to produce the feature map. The advantage of such kernel sharing is that it can reduce the model complexity and makes network training easier.

The different kernels produce complete feature maps. The $k$-th feature map at $\left ({i,j}\right)$ location of the $l$-th layer, $h_{i,j,k}^{l}$, is calculated as:

\begin{equation*} {h_{i,j,k}^{l}}={W_{k}^{l}{}^{T}}{X_{i,j}^{l}}+{b_{k}^{l}}\tag{6}\end{equation*}

where $X_{i,j}^{l}$ is input value centered at location $\left ({i,j}\right)$ of the $l$-th layer, and $W_{k}^{l}$ and $b_{l}^{k}$ are the weight vector and bias term of the $k$-th filter of the $l$-th layer, respectively.

Detection of nonlinear features of the input, which are desirable for multi-layer networks, is achieved through the use of the activation function. The nonlinear activation function, denoted by $a\left ({.}\right)$ produces the activation value $a_{i,j,k}^{l}$ of the convolutional feature $h_{i,j,k l}$ as–

\begin{equation*} a_{i,j,k}^{l} = a\left ({{h_{i,j,k}^{l}}}\right)\tag{7}\end{equation*}

Pooling layers are used to reduce the volume of the feature map by aggregating small rectangular subsets of values. Two types of pooling, namely, Max and Average, are applied to replace the input values with the maximum or the average value, respectively. The output of the pooling function $p\left ({.}\right)$ for each feature map $a_{i,j,k}^{l}$ is:

\begin{equation*} g_{i,j,k}^{l} = p\left ({{a_{m,n,k}^{l}}}\right),\quad \forall {\left ({m,n}\right)} \in R_{i,j}\tag{8}\end{equation*}

where $R_{ij}$ is a local neighbourhood around location $\left ({i, j}\right)$.

Classification networks on top of the convolutional/pooling layers typically contain a set of sequential fully connected layers, and consist of nodes with various activation functions. Fully connected layers are typically used as the last few layers of the model. The output of the last pooling layer is flattened and fed to the feed-forward neural network for classification of the inputs. Just following the fully connected layers, a classifier is used to calculate the probability of each instance belonging to each class. The last layer is designed to have as many outputs as labels. The output layer is softmax activated. For a given input sample $X$, the softmax function predicts the probability for the $c$th class as:

\begin{equation*} O_{W,b}{\left ({X}\right)}= P(y=c|X;W,b)=\frac {\exp ^{X^{T}W_{c}}}{\sum _{c=1}^{C}{\exp ^{X^{T}W_{c}}}}\tag{9}\end{equation*}

where $c$ is the current class being evaluated, $C$ is all classes, $X$ is the input vector, and $W$ represents network weights.

#### 1) ResNet50

We have also used Residual Neural Network (ResNet) to compare the classification results. ResNet was first introduced at the 2015 ILSVRC competition by He *et al.* [29]. Resnet has a short connection structure that is used to prevent the problem of gradient vanishing by bypassing the input information directly to the output. ResNet is a network-in-network (NIN) architecture that consists of stacking many residual modules. These residual units are used to build deep ResNet50 architecture. The residual units consist of convolution, pooling, and layers. ResNet50 uses global average pooling instead of fully connected layers. We adopted and customized the ResNet50 [29] deep CNN architecture by removing the fully connected and output layers and adding two fully connected layers; a dropout layer, and an output layer for two classes. We used the same dense layer network after the convolutional layers as in the 4L-CNN.

#### 2) VGG16

The Visual Geometry Group (VGG) network architecture was initially proposed by Simonyan and Zisserman [30] in 2014 for the ImageNet Challenge competition. The adopted classification model based on VGG16 consists of 16 convolutional layers, one averaging pooling layer, two dense layers, one dropout layer, and the output layer.

The VGG16 architecture consists of five blocks of convolutional layers and some fully-connected layers. To retain the same spatial dimension of the feature maps between layers, a $3\times3$ kernel, stride size 1, and padding of 1 is used in the convolutional layers. The spatial dimension of the feature maps is reduced by using a rectified linear unit (ReLU) activation function just after the convolutional layer, and by performing a max-pooling operation after the end of each block. A max pooling layer with a $2\times2$ kernel and stride size of 2 is used to make the spatial dimension of the activation map half the previous layer.

### D. Proposed Methodology

We propose three methods of EEG seizure classification based on adopted CNN models. In the first method, Method-1 (4L-CNN), we train a CNN-based model from scratch. In Method-2 (transfer learning only or TL), we freeze the pre-trained deep CNN from training and train only top dense layers using the output features of the pre-trained networks. Method-3 (Fine-tuning or FT) involves two steps; first fine-tuning the top dense layers is performed using the output features of the pre-trained networks, then the deep bottom layers (deep CNN model) are fine-tuned by initializing the pre-trained weights on ImageNet.

#### 1) Method-1 (4L-CNN): Training a Shallow Model From Scratch

**FIGURE 1.**
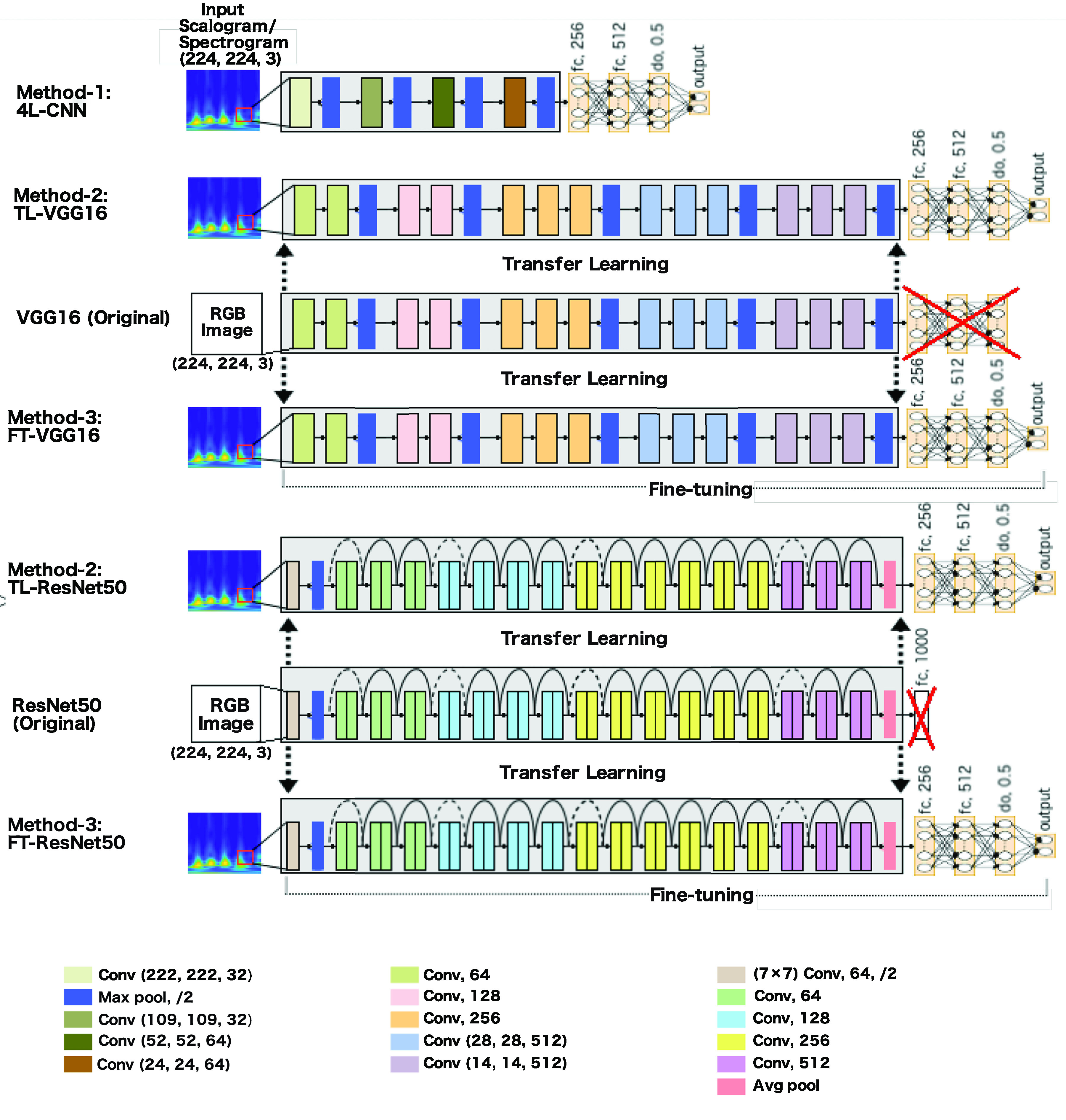
The proposed methods for seizure classification. 4L-CNN in Method-1 is a shallow classifier built with four convolutional layers. In Method-2 and Method-3, VGG16 and ResNet50 are adopted to classify seizures from normal EEG. In Method-2, a transfer learning technique is used to load the weights of the convolutional part of the network, and features are extracted from the spectrogram/scalogram input; then the extracted features are fed into fine-tuned dense layers. In Method-3, both the convolutional and dense layers are fine-tuned.

Method-1 involves training a CNN-based shallow model for seizure classification from scratch, shown in Fig. 1. The simple four convolutional layers CNN (4L-CNN) consists of 4 convolutional layers, two fully connected layers, a dropout layer, and an output layer for classification of seizure or non-seizure. After four sequential convolutional layers, four fully connected layers are used to build a dense layer network. The first fully connected layer consists of 256 ReLU activated nodes, and receives the flattened output of the convolutional part of the network. The second fully connected layer is also ReLU activated and contains 512 neurons receiving a 256 dimensional vector from the output of the previous layer. The third fully connected layer consisting of 512 nodes, is a dropout layer with 50% drop out. Overfitting is avoided by using dropout, which randomly ignores some neurons in training. Finally, the output of the dropout layer is fed into a softmax activated output that assigns a probability for each class.

#### 2) Method-2 (TL): Training the Top Dense Layers Only of the Adopted Network

In Method-2 (TL), the transfer learning technique is used to load the weights of the model. The time-frequency images of the EEG are provided as input to the pre-trained modified deep CNN model to get the output features. These output features, obtained from the last layer of the deep CNN model before the FC layer, are then used to train only the fully connected layers. The architecture of the fully connected layers is the same as the top FC layer described in Method-1 (4L-CNN). In this method, shown in Fig. 1, VGG16 and ResNets are loaded.

#### 3) Method-3 (Fine-Tuning or FT): Fine-Tuning the Adopted Networks in Two Steps

In Method-3 (Fine-tuning or FT), the convolutional layers of the loaded deep CNN model (VGG16 or ResNet50) (shown in Fig. 1) are fine-tuned together with the top-level FC layers. Because of differences between images in ImageNet and the time-frequency represented images of the EEG, we have fine-tuned all layers with the intention of increasing accuracy. Fine-tuning is an advanced practice of transfer learning. This method is implemented in a few steps. First, build the top layers of the network using a deep CNN model, and load the pre-trained weights of the model on ImageNet. Second, freeze the convolutional and other layers up to the first FC layer and train only the last few FC layers using the extracted features from the deep CNN; just like the model training in Method 2. Lastly, we freeze the FC layers and train only the deep CNN model.

Throughout this paper we will refer to the CNN-based shallow model in method-1 as 4L-CNN, the VGG16-based model in Method-2 as TL-VGG16, the ResNet50-based classifier in Method-2 as TL-ResNet50, the VGG16-based model in Method-3 as FT-VGG16, and the ResNet50-based model in Method-3 as FT-ResNet50.

### E. Feature Importance Calculation Using SHAP

The evaluation metric in terms of accuracy, sensitivity, and specificity, does not always give us a complete picture of how the classification decision was made. When a classification model is tested, we sometimes are interested in an explanation as to why the output is made; that is, which input features are mostly responsible for this decision making. In addition, knowing more about the classification can help us to learn more about the data. Although explaining the outputs of deep learning models is often challenging, the SHapley Additive exPlanations (SHAP) [25] tool can help us interpret the outputs in terms of feature importance. SHAP provides a way to estimate the contribution of each feature to the output of the model.

The integrated gradients method calculates the importance score of a feature value $i$ as:

\begin{align*} \phi _{i}^{IG}{(f,x,{x'})} = \left ({x_{i}-x_{i}'}\right)\times \int _{\alpha =0}^{1} \frac {\delta {f\left ({x'+\alpha \left ({x-x'}\right)}\right)}}{\delta {x_{i}}}d\alpha \\\tag{10}\end{align*}

where $x'$ is some arbitrary baseline input, $x$ is the present input, $f$ is the model function.

From the equation 10 we see that it accumulates gradients on images interpolated between the present image and the baseline image. However, calculating feature importance using gradients suffers from thresholding. A novel feature attribution method, called Expected Gradients [31] is used to calculate the SHAP values. Gradient SHAP is also known as the Expected gradient is an upgradation of integrated gradients [32] which explains the difference between the model’s prediction with an arbitrarily chosen reference input (baseline input), and it’s current prediction. Expected gradient methods avoid using arbitrary reference inputs. An underlying training data distribution is used to calculate the reference input.

## III. Experimental Setups and Results

This section focuses on the experimental setup and presentation of the results. First, we describe the experimental setup namely, the system configuration and the implementation details.

### A. Experimental Setup

Herein we use a Windows gaming computer with Intel(R) Core (TM) i7-7700HQ (2.80 GHz, 2808 Mhz, 4 Cores and 8 Logical processors) CPU, 16 GB RAM, an NVIDIA GeForce GTX 1060 6GB Graphical Processing Unit (GPU), and CUDA 9.0 for GPU acceleration running on a Windows 10 64-bit system. Matlab (R2018a) and Python programming languages were used to conduct the experiments.

EEG segmentation and time-frequency representation were performed using Matlab. The model training and testing, and model decision explanation were performed using the Keras [33] library.

To combat overfitting, we adopted two strategies. The first is data augmentation, and the second is a dropout. The simplest and most popular method of minimizing overfitting on image data is to enlarge the dataset using label-preserving transformations artificially [34]. Data augmentation produces transformed images from the original images with very simple computation, and augmented images do not need to be stored on disk. In this study, we augmented the images by translations to reduce test errors. Another strategy we follow to combat the overfitting problem is the dropout technique [35]. Dropout sets the probability 0.5 to each hidden neuron to produce zero output. In this way, the neurons that are “dropped out” do not contribute to the forward and backward pass during the training. We use dropout in the layer just before the output layer. We used the Root Mean Square Propagation optimizer (RMSprop) [36] with a batch size of 10 samples and learning rate 0.00001. We rescaled the images to $224\times 224$ dimensions and each input has 3 (RGB) channels.

**FIGURE 2.**
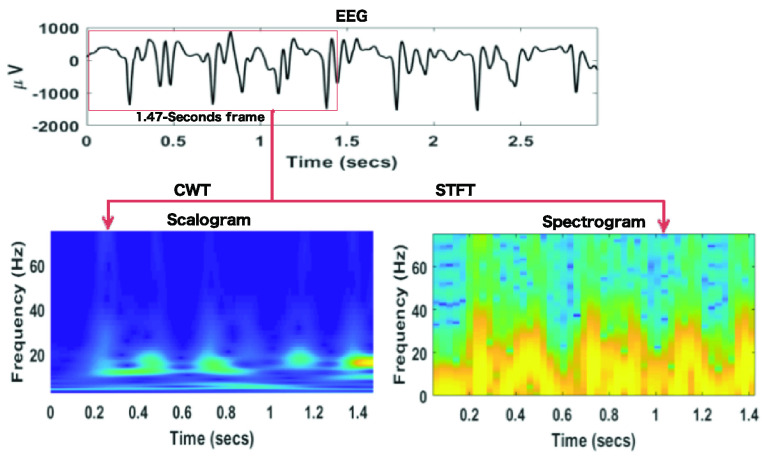
Prepraring spectrogram and scalogram for a 1.47 seconds EEG segment. The frame is moving without overlapping to get a new spectrogram and scalogram image.

In this study, all five groups of the EEG datasets (A, B, C, D, E) have been used. In all the proposed methods, the first step is to obtain the time-frequency (t-f) represented images from the EEG signal using both signal-to-image conversion techniques, STFT and CWT. Fig. 2 shows the STFT spectrogram and CWT scalogram from a segment of EEG signals. Once the production of the t-f images is completed, they are split into training, validation, and test sets. We have 1600 t-f images for each dataset. The training dataset contains 80% of total images, the testing dataset contains 10% of total images and validation datasets contain 10% of the total images. The same training, validation, and test sets are used in all the proposed methods for training, validation, and testing the classifiers. All the experimental cases studied are shown in Table 1 and they deal with binary classification. A total of fifteen binary cases are tested in order to classify seizures from normal EEG. We compared results in terms of accuracy, sensitivity, and specificity obtained from the proposed methods.

**TABLE 1:** Different Cases Studied in This Work

Cases	Description	
	Class 1	Class 2
A_E	Normal (eyes open)	Seizure
B_E	Normal (eyes close)	Seizure
C_E	Inter-ictal	Seizure
D_E	Inter-ictal	Seizure
AB_E	Normal	Seizure
AC_E	Normal (eyes open) and Inter-ictal	Seizure
AD_E	Normal (eyes open) and Inter-ictal	Seizure
BC_E	Normal (eyes close) and Inter-ictal	Seizure
BD_E	Normal (eyes close) and Inter-ictal	Seizure
CD_E	Inter-ictal	Seizure
AB C_E	Normal and Inter-ictal	Seizure
ABD_E	Normal and Inter-ictal	Seizure
ACD_E	Normal (eyes open) and Inter-ictal	Seizure
BCD_E	Normal (eyes close) and Inter-ictal	Seizure
ABCD_E	Normal and Inter-ictal	Seizure

### B. Results

**FIGURE 3.**
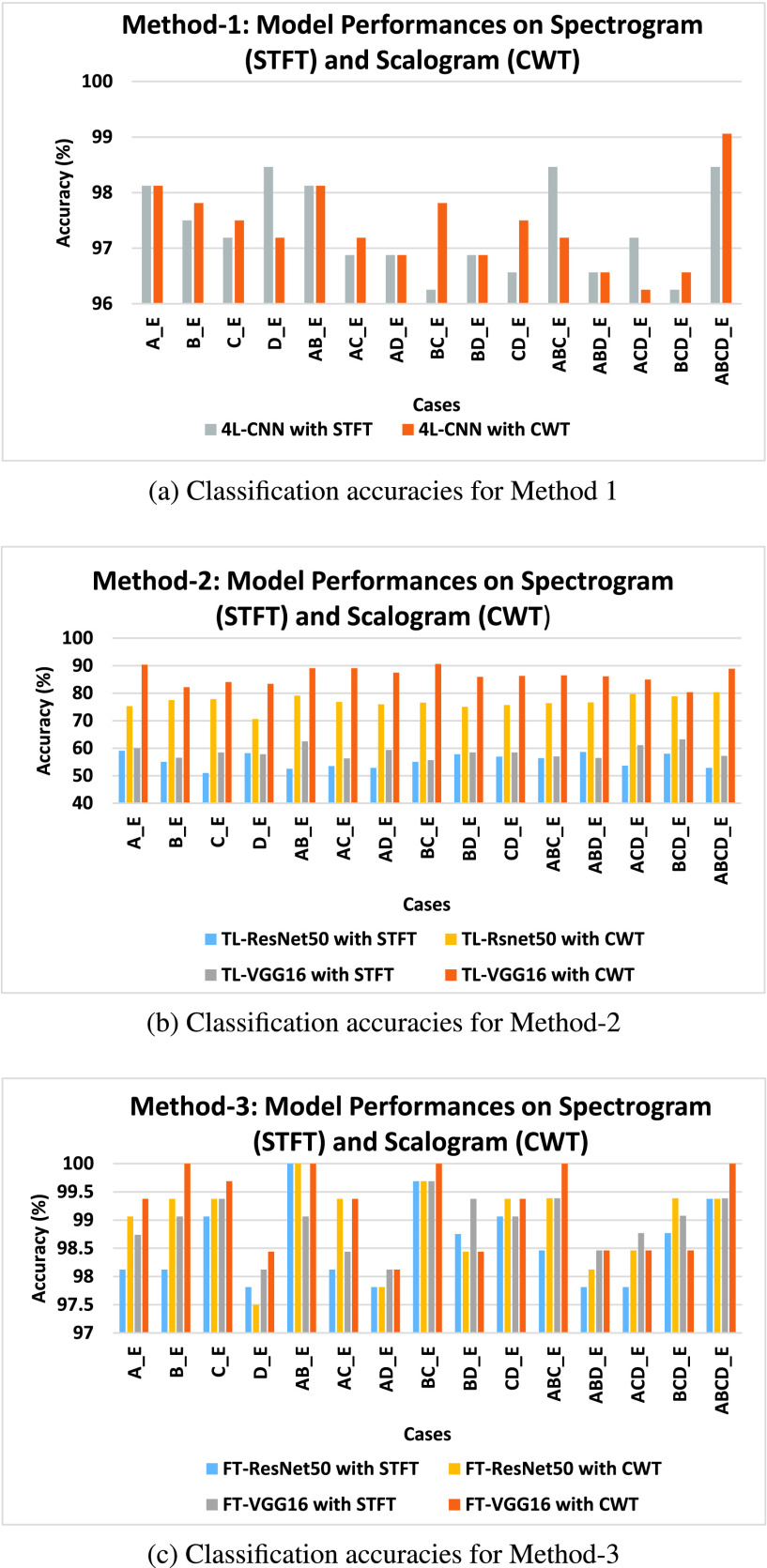
Comparison of classfication accuracy obtained from the proposed methods using both STFT scalogram and CWT scalogram for 15 binary cases. a) The results obtained from the 4L-CNN classifier in Method-1 using both STFT spectrogram and CWT scalogram. b) The classification accuracy obtained from the classifers TF-VGG16 and TF-ResNet50 in Method-2 using using both STFT spectrogram and CWT scalogram. c) The classification accuracy for seizure and non-seizure EEG classification using FT-VGG16 and FT-ResNet50 models in Method-3 and spectral images.

First, we evaluated the performance of the proposed signal-to-image conversion methods for seizure classification. In Fig. 3, the classification accuracy for all the cases obtained from the proposed methods are presented. The distinguishing ability of the epileptic and normal EEG is well represented in CWT scalogram, showing better results than the STFT spectrogram. For Method-1, 7 cases (B_E, C_E, AC_E, BC_E, CD_E, BCD_E, and ABCD_E) showed better results for CWT scalogram than STFT spectrogram, 5 cases (A_E, AB_E, AD_E, BD_E, and ABD_E) showed equal classification accuracy, and only 3 cases (D_E, ABC_E, and ACD_E) STFT spectrogram performed better than CWT scalogram (shown in Fig. 3a). In Fig. 3b, the classification accuracy of Method-2 using the CWT scalogram and STFT spectrogram is shown. We have computed the classification accuracy for the two proposed models, TL-VGG16 and TL-ResNet50, and obtained the highest results of all the cases for the CWT scalogram. It is observed from Fig. 3c that the classification accuracy in Method-3 is higher for the CWT scalogram for most of the cases. The FT-ResNet50 classifier proposed in Method-3 obtains highest results for CWT scalogram than STFT spectrogram for 9 cases (A_E, B_E, C_E, AC_E, CD_E, ABC_E, ABD_E, ACD_E, and BCD_E) out of 15 cases and equal accuracy for 4 cases (AB_E, AD_E, BC_E, and ABCD_E). The FT-VGG16 in Method-3, shows the highest accuracy for CWT scalogram compared to the STFT spectrogram for 10 cases (A_E, B_E, C_E, D_E, AB_E, AC_E, BC_E, CD_E, ABC_E, and ABCD_E) and equal accuracy for both signal-to-image conversion techniques for 2 cases (AD_E and ABD_E).

**FIGURE 4.**
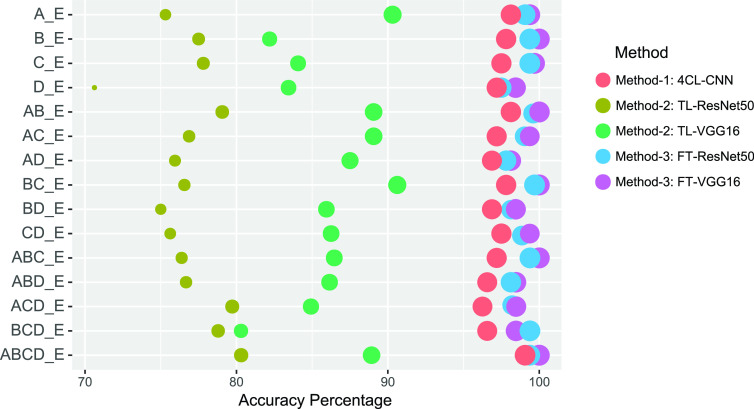
Bubble plot showing the overall classification accuracy (%) obtained from all five classifiers in the proposed methods using CWT scalogram. Results show the FT-CGG16 with CWT scalogram perform the best than all the other classifiers.

Given the CWT scalogram method performed better than STFT spectrogram in classification, we thus considered the experiments which use only CWT scalogram as input. Fig. 4 shows the classification accuracy of the classifiers of all the proposed methods using CWT scalogram. The FT-VGG16 classifier proposed in Method-3 classified seizure EEG with the highest accuracy in most cases (A_E, B_E, C_E, D_E, AD_E, BC_E, ABC_E, ABD_E, and ABCD_E) compared to FT-ResNet50 in Method-3 and classifiers in other Methods. In some cases (AB_E, AC_E, BD_E, CD_E, and ACD_E), FT-VGG16 and FT-ResNet50 classifiers proposed in Method-3 show identical results in terms of classification accuracy. Overall the FT-VGG16 classifier using CWT scalogram performs the best among all the proposed classifiers.

**FIGURE 5.**
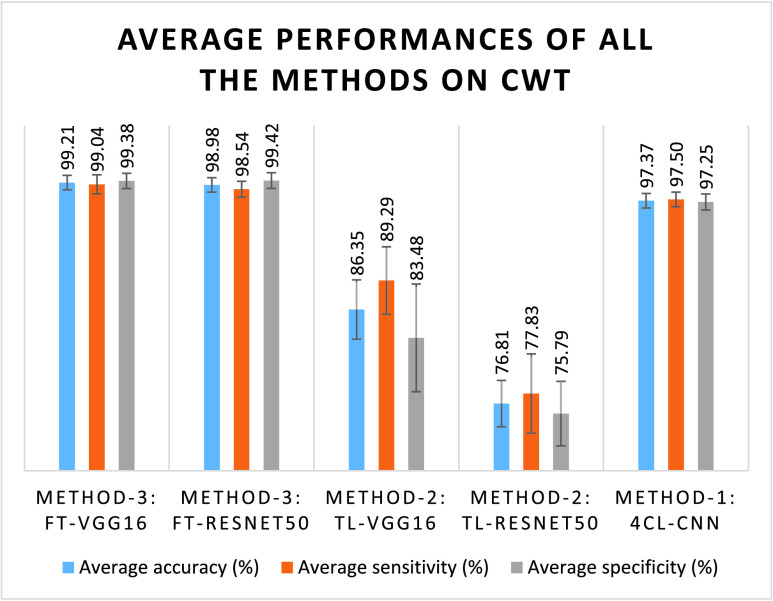
Bar graphs of the average classification performances over 15 cases of all the methods using CWT scalogram.

In Fig. 5, the average classification accuracy, sensitivity, and specificity for all the classifiers using the CWT scalogram are presented. This shows that the performance of Method-3 is very promising compared to other methods. The proposed FT-VGG16 classifier in Method-3 achieves the highest average classification accuracy (99.21%) with the highest average sensitivity (99.04%). The average specificity obtained by FT-VGG16 classifier in Method-3 is slightly lower, 99.38%, than the average specificity obtained from FT-ResNet50 classifier in Method-3 that is 99.42%. The average performance of FT-VGG16 classifier in Method-3 is very promising as compared to the FT-ResNet50 classifier. In Table 2, we have shown the classification performance in terms of accuracy, sensitivity, and specificity using our proposed FT-VGG16 classifier and CWT scalogram. The FT-VGG16 obtained the highest classification accuracy of 100% for the classification cases B_E, AB_E, BC_E, ABC_E, and ABCD_E, the highest sensitivity of 100% for the A_E, B_E, AB_E, BC_E, ABC_E, and ABCD_E cases, and the highest specificity of 100% for the classification cases, namely B_E, C_E, AB_E, AC_E, BC_E, CD_E, ABC_E, and ABCD_E. The classification performances using the proposed CWT scalogram and FT-VGG16 of Method-3 for other cases are also noteworthy. The classification accuracy of 99.38%, 99.69%, 98.44%, 99.38%, 98.13%, 98.44%, 99.38%, 98.46%, 98.46%, and 98.46% for the cases of A_E, C_E, D_E, AC_E, AD_E, BD_E, CD_E, ABD_E, ACD_E, and BCD_E, respectively, are achieved using the proposed FT-VGG16 classifier and CWT scalogram.

**TABLE 2:** Classification Measures of the Proposed FT-VGG16 Classifier in Method-3 Using CWT Scalogram

Cases	Accuracy (%)	Sensitivity (%)	Specificity (%)
A_E	99.38	100.00	98.75
B_E	100.00	100.00	100.00
C_E	99.69	99.38	100.00
D_E	98.44	97.50	99.38
AB_E	100.00	100.00	100.00
AC_E	99.38	98.75	100.00
AD_E	98.13	97.50	98.75
BC_E	100.00	100.00	100.00
BD_E	98.44	98.75	98.13
CD_E	99.38	98.75	100.00
ABC_E	100.00	100.00	100.00
ABD_E	98.46	98.75	98.18
ACD_E	98.46	97.50	99.39
BCD_E	98.46	98.75	98.18
ABCD_E	100.00	100.00	100.00

**FIGURE 6.**
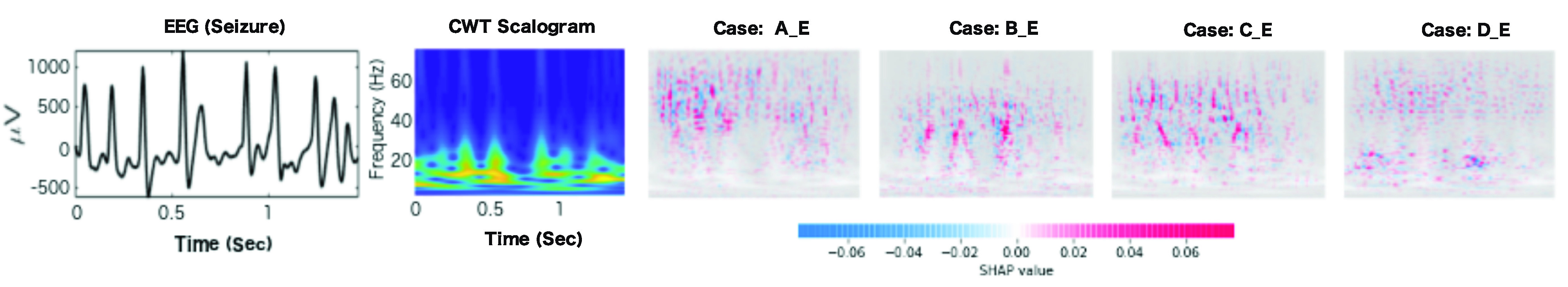
The SHAP value shows the important frequency features in the input scalogram of seizure EEG. The seizure EEG is shown on the left, followed by the corresponding CWT scalogram. The SHAP value plotted (red) in the corresponding grayscale image of the CWT scalogram show the important frequency features of the scalogram that increased the classifier’s prediction for each case.

Fig. 6 shows the important features of the scalogram in terms of SHAP values which are considered to be the significant frequency components in the seizure EEG that play key roles in classification decision making. We considered four cases, namely A_E, B_E, C_E, and D_E for identifying important frequency features in the seizure EEG. Frequency components within 40Hz to 60Hz range in seizure EEG were important for being distinct to normal (eyes open) EEG. The SHAP values were high in the input scalogram of seizure EEG for the frequencies ranged from 30Hz to 40Hz, for the case B_E. For the case C_E, frequency components within the 30Hz to 60Hz range in the seizure scalogram were scored the highest. The SHAP values were distributed in the seizure EEG scalogram in two spectrum ranges, from 10Hz to 25Hz and 40Hz to 60Hz, for the D_E case. This indicates that these two ranges of frequency components were significant for the seizure scalogram, to be distinguishable from the EEG of dataset D.

## IV. Discussion

Electroencephalography (EEG) is used to capture the electrical activity of the brain. We wanted to compare the applicability of two widely used time-frequency representations of EEG signal, STFT and CWT, in a deep learning model for EEG classification purposes. For time-frequency representations of the EEG we have obtained time-frequency images, spectrograms for STFT, and scalograms for CWT. We classified seizure and non-seizure EEG signals in this study and demonstrate that classification using CWT-based scalogram outperformed the STFT-based spectrogram in almost all cases.

The poorer performance of the STFT spectrogram limits its applicability in EEG epilepsy classification. The lower performance of STFT can be characterized by its fixed frequency resolution and determined by the fixed length of the analysis window. The fixed width of the window function gives rise to a fixed frequency resolution. The STFT fails to capture some critical time-frequency information in spectrograms due to the poorer time-frequency resolution which provides poor input images for the deep learning model. However, in CWT, the window length or dilation parameter is dependent on the frequency component being measured which produces better time-frequency resolution. Thus, we propose the CWT scalogram input and the FT-VGG16 classifier in Method-3 for the EEG epilepsy classification tasks. For the EEG classification of seizures in all cases, the average classification accuracy we report is 99.21% using the FT-VGG16 model and CWT scalogram images; compared with 98.94% for the STFT spectrogram approach. Additionally, evaluating the two proposed classifiers in Method-3, we observed that the adopted fine-tuned VGG16-based classifier (FT-VGG16) achieved higher classification accuracy than FT-ResNet50.

Results comparison between the proposed FT-VGG16 model in Method-3 in the present study, and existing studies [2], [6]–[8], [10], [11], [37], [38] is shown in Table 3. The results reported in the existing studies used for comparison are published within the last ten years and we compared with only those studies that obtained at least 95% classification accuracy. In the existing literature, many of the current studies have considered only a few experimental cases (A_E, B_E, C_E, D_E, and ABCD_E etc.). In contrast, we have considered 15 independent experimental cases to validate our approach.

**TABLE 3:** Comparison of Classification Accuracy Obtained by Our Proposed Approach (Method 3: VGG16) Compared to the Classification Accuracy Obtained by the Different Existing Studies

Cases	Authors	Year	Method	Results		
				Accuracy (%)	Sensitivity (%)	Specificity (%)
A_E	[10]	2011	Extreme learning machine (ELM)	96.50	92.50	96.00
A_E	[2]	2016	SVM	97.25	94.50	100.00
A_E	[37]	2017	ANN and LNDP	99.82	99.90	99.75
A_E	[7]	2018	Deep Pyramidal 1D-CNN	100.00	–	–
A_E	[8]	2019	ESD-LSTM	100.00	100.00	100.00
A_E	[38]	2019	CNN and CWT	99.50	99.00	100.00
A_E	[6]	2020	1D-CNN	99.52	–	–
A_E	–	–	Proposed FT-VGG16 and CWT	99.38	100.00	98.75
B_E	[37]	2017	ANN and LNDP	99.25	99.10	99.40
B_E	[7]	2018	Deep Pyramidal 1D-CNN	99.80	–	–
B_E	[38]	2019	CNN and CWT	99.50	100.00	100.00
B_E	[6]	2020	1D-CNN	99.11	–	–
B_E	–	–	Proposed FT-VGG16 and CWT	100.00	100.00	100.00
C_E	[2]	2016	SVM	96.00	95.83	96.15
C_E	[37]	2017	ANN and 1D-LGP	99.10	98.75	99.45
C_E	[7]	2018	Deep Pyramidal 1D-CNN	99.10	–	–
C_E	[38]	2019	CNN and CWT	98.50	98.01	98.98
C_E	[6]	2020	1D-CNN	98.02	–	–
C_E	–	–	Proposed FT-VGG16 and CWT	99.69	99.38	100.00
D_E	[37]	2017	ANN and 1D-LGP	99.07	98.82	99.32
D_E	[7]	2018	Deep Pyramidal 1D-CNN	99.40	–	–
D_E	[38]	2019	CNN and CWT	98.50	98.01	98.98
D_E	[6]	2020	1D-CNN	97.63	–	–
D_E	–	–	Proposed FT-VGG16 and CWT	98.44	97.50	99.38
AB_E	[7]	2018	Deep Pyramidal 1D-CNN	99.80	–	–
AB_E	[6]	2020	1D-CNN	99.38	–	–
AB_E	–	–	Proposed FT-VGG16 and CWT	100.00	100.00	100.00
AC_E	[6]	2020	1D-CNN	99.03	–	–
AC_E	–	–	Proposed FT-VGG16 and CWT	99.38	98.75	100.00
AD_E	[6]	2020	1D-CNN	98.50	–	–
AD_E	–	–	Proposed FT-VGG16 and CWT	98.13	97.50	98.75
BC_E	[7]	2018	Deep Pyramidal 1D-CNN	99.50	–	–
BC_E	[6]	2020	1D-CNN	98.68	–	–
BC_E	–	–	Proposed FT-VGG16 and CWT	100.00	100.00	100.00
BD_E	[6]	2020	1D-CNN	97.83	–	–
BD_E	–	–	Proposed FT-VGG16 and CWT	98.44	98.75	98.13
CD_E	[11]	2017	Random forest and correletion based feature selection	98.67	98.70	98.70
CD_E	[37]	2017	ANN and LNDP	98.88	97.05	99.80
CD_E	[6]	2020	1D-CNN	98.03	–	–
CD_E	–	–	Proposed FT-VGG16 and CWT	99.38	98.75	100.00
ABC_E	[7]	2018	Deep Pyramidal 1D-CNN	99.97	–	–
ABC_E	[6]	2020	1D-CNN	98.89	–	–
ABC_E	–	–	Proposed FT-VGG16 and CWT	100.00	100.00	100.00
ABD_E	[6]	2020	1D-CNN	98.52	–	–
ABD_E	–	–	Proposed FT-VGG16 and CWT	98.46	98.75	98.18
ACD_E	[11]	2017	Random forest and correletion based feature selection	98.50	98.50	98.50
ACD_E	[7]	2018	Deep Pyramidal 1D-CNN	99.80	–	–
ACD_E	–	–	Proposed FT-VGG16 and CWT	98.46	97.50	99.39
BCD_E	[11]	2017	Random forest and correletion based feature selection	97.50	97.50	97.50
BCD_E	[6]	2020	1D-CNN	98.36	–	–
BCD_E	–	–	Proposed FT-VGG16 and CWT	98.46	98.75	98.18
ABCD_E	[11]	2017	Random forest and correletion based feature selection	97.40	97.40	97.50
ABCD_E	[37]	2017	ANN and LNDP	98.72	98.30	98.82
ABCD_E	[7]	2018	Deep Pyramidal 1D-CNN	99.70	–	–
ABCD_E	[8]	2019	ESD-LSTM	100.00	100.00	100.00
ABCD_E	[6]	2020	1D-CNN	98.76	–	–
ABCD_E	–	–	Proposed FT-VGG16 and CWT	100.00	100.00	100.00

Case A_E shows the classification accuracy of 99.38% obtained from the proposed FT-VGG16 approach; the highest result with perfect accuracy of 100% is achieved in [7], [8] using Deep Pyramidal 1D-CNN, and ESD-LSTM, respectively. For this A_E case, methods using extreme learning machine (ELM) [10] and SVM [2] achieved a classification accuracy of 96.50% and 97.25% respectively, which are lower than the classification accuracy gained by our proposed FT-VGG16.

For case B_E this study achieved a perfect classification accuracy of 100% using FT-VGG16 and CWT, which is the highest compared with other studies with a classification accuracy of 99.25% using ANN and LNDP [37], 99.80% using Deep Pyramidal 1D-CNN [7], 99.50% using CNN and CWT [38], and 99.11% using 1D-CNN [6].

For the case C_E, the classification accuracy obtained from our proposed FT-VGG16 classifier is 99.69% which is the maximum compared to previous studies [2], [6], [7], [37], [38] with the reported accuracy of 96.00%, 99.10%, 99.10%, 98.50%, and 98.02%, respectively. It is shown that for the case D_E, compared to the maximum result obtained in other studies [7] using deep pyramidal 1D-CNN and [37] using ANN and 1D-LGP, however the present study achieved the highest classification accuracy including in comparision to the results obtained using CNN and CWT [38]), and using 1D-CNN [6].

In case AB_E, this study achieved the classification accuracy of 100% with FT-VGG16 which is the best obtained for this data set. For the same case, the current results also reported using 1D-CNN [7], and using 1D-CNN [6] with accuracies 99.80% and 99.38% respectively.

For experimental case AC_E, we have obtained 99.38% classification accuracy. We have not found many studies that conducted an experiment for this case; however, recently, Zhao *et al.* [6] reported classification accuracy of 99.03% for the same case AC_E which is 0.35% less than the classification accuracy obtained with the proposed FT-VGG16 and CWT. In case AD_E, the results obtained from the proposed FT-VGG16 classifier in Method-3 is 98.13% which is 0.37% less than obtained in [6].

For the seizure recognition cases BC_E, BD_E, and CD_E, the FT-VGG16 obtained the best performances with the accuracy of 100%, 98.44%, and 99.38% respectively, than the conventional methods. In the literature, there are very few studies that address classification problems associated with ABC_E, ABD_E, ACD_E, and BCD_E cases classification problems. The proposed FT-VGG16 also achieved good classification performances for those types of cases. Finally, the proposed FT-VGG16 achieved 100% accuracy for the ABCD_E case. Shallow machine learning model including SVM was also used to classify epilepsy by using the same dataset and DWT based fuzzy approximate entropy, and we obtained classification accuracy greater than 95% [39]. SMV and weighted-permutation Entropy approach in [2] achieved an average epilepsy detection accuracy of 91.62 for six different cases. Another study [40] also used weighted-permutation Entropy and obtained classification accuracy 91.65% and 93.75% for linear and non-linear SVM, respectively. Although for all the cases the proposed FT-VGG16 did not achieve the highest accuracy compared to the existing studies, we can consider it useful as it showed good classification accuracy for most of the cases.

To the best of our knowledge, this is the first study that analysed the important and responsible frequency components in seizure EEG for classification by explaining the gradients of the deep learning model. The SHAP values obtained from the FT-VGG16 model showed that mainly the higher frequency components in EEG seizures are significant to the classifier’s correct prediction. The proposed deep learning model can be deployed in real-world clinical practices in automatic epileptic seizure detection as it showed very high classification accuracy. Both clouds based and the stand-alone diagnostic system could be developed using the proposed model. The characteristics frequency in EEG found using SHAP could be useful to the clinicians to better interpretation and understanding of epilepsy in EEG.

## V. Conclusion

First, the proposed methods presented in this paper accurately classify EEG epilepsy. From the five EEG datasets (A, B, C, D, and E), a total of 15 cases of binary classification have been tested. The time-domain EEG signals have been converted to time-frequency images with the aim to detect the seizure (ictal) from the normal, inter-ictal EEGs accurately. Two different types of signal-to-image conversion techniques, STFT and CWT, have been applied, and they show the differences in classifier performance. The CWT scalogram has been chosen as the better time-frequency representation of the EEG signal for this seizure classification problem as it shows better performances than the STFT spectrogram. Among the five classifiers in three proposed methods, the FT-VGG16 in Method-3 with CWT scalogram provides better classification results, with an average accuracy of 99.21%, the sensitivity of 99.04%, and specificity of 99.38% of 15 cases. The proposed FT-VGG16 with CWT is compared with the existing methods. For most of the cases, the proposed FT-VGG16 shows the highest accuracy compared with conventional methods in the existing literature. Additionally, frequency bands that contribute most to predictive accuracy in seizure EEG using the FT-VGG16 classifier have been identified. The higher frequencies in seizure EEG are more significant for the classifier to correctly predict the seizure EEG from normal and inter-ictal EEG. To the best of our knowledge, this is the first study of its kind study using CWT based time-frequency representation of EEG and a very deep CNN model for seizure detection, as well as identifying characteristic frequencies that enable accurate automated EEG seizure prediction.
